# Cocaine bugs: A brief case report of cocaine-induced delusion of parasitosis

**DOI:** 10.1192/j.eurpsy.2021.1708

**Published:** 2021-08-13

**Authors:** J. Quarenta, S. Martins, T. Teixeira, J.P. Ribeiro

**Affiliations:** 1 Psychiatry Department, Centro Hospitalar entre o Tamega e Sousa EPE, Penafiel, Portugal; 2 Departamento De Psiquiatria E Saúde Mental, Centro Hospitalar Tâmega e Sousa, Penafiel, Portugal

**Keywords:** Ekbom, Delusional parasitosis

## Abstract

**Introduction:**

Delusional parasitosis (DP), also know as Ekbom syndrome and in some cases as Morgellons, was first described in the late 17th century in France. It is an obsessive phobic state in which the patient believes that the is infested by parasites. In the hallucinatory state, they frequently remove parts of the skin, identifying them as parasites. The cause of DP is unknown. Evidence supporting the dopamine theory defend that the inhibition of dopamine reuptake (for example cocaine and amphetamines) induce symptoms such as formication.

**Objectives:**

Through the description of the following clinical case, we emphasize its clinical features and complexities.

**Methods:**

Review of DP in light of a clinical case

**Results:**

A 48-year-old woman was brought to the psychiatric emergency due to psychotic symptoms following cocaine use. She had a history of drug abuse. She was apparently asymptomatic until October 2019, when, in the background of vague sensation of something crawling under his skin, she developed a sudden onset belief that she had been infested by insects that crawled under his skin. Previous medical observation found no reason for a skin infection or infestation. Skin examination revealed itch marks and skin excoriations in the abdomen. Mental status examination revealed anxious and depressive affect, delusion of parasitosis, tactile hallucination and impaired insight. Routine hemogram and urinalysis was unremarkable, except for the detection of cocaine.

**Conclusions:**

Delusional parasitosis often presents to nonpsychiatric medical professionals. An awareness of such ilness, with an early recognition and timely referral are management cornerstones in order to successfully diagnose and treat patients.

**Disclosure:**

No significant relationships.

